# Understanding How a Digital Platform for Chronic Disease Management Can Enable and Limit Patient Self-Care: Qualitative Study

**DOI:** 10.2196/71875

**Published:** 2026-05-14

**Authors:** Lysanne Lessard, Mark de Reuver, Kerri-Anne Mullen

**Affiliations:** 1 Telfer School of Management, University of Ottawa, Ottawa, ON, Canada; 2 Institut du Savoir Montfort, Ottawa, ON, Canada; 3 Engineering Systems and Services, Technology Policy and Management, Delft University of Technology, Postbus 5, 2600 AA, Delft, The Netherlands, 31 15 27 81920; 4 Ottawa Heart Institute, Ottawa, ON, Canada; 5 School of Epidemiology and Public Health, Faculty of Medicine, University of Ottawa, Ottawa, ON, Canada

**Keywords:** digital platform, chronic disease management, self-care, qualitative study, self-management, patient experience

## Abstract

**Background:**

A growing segment of the population requires ongoing care and support for managing their chronic diseases. Digital platforms for self-management are rapidly emerging to meet this need, but patients’ experiences with these platforms vary significantly. This may be due to the complexity and flexibility of digital platforms, where the wide array of available features can generate unexpected impacts.

**Objective:**

This study aims to explore how a digital platform can both enable and limit patients with a chronic disease in managing their own health.

**Methods:**

We conducted semistructured qualitative interviews with patients to better understand their experience of using a digital platform for self-managing their chronic diseases. Patients who had been using a digital platform (the Chronic Care Platform) for at least 1 month were invited to participate. Twenty-four patients were recruited and interviewed in person or by phone. The collected data were analyzed using template analysis, which is a type of thematic analysis that allows inductive identification of themes from data and deductive application of theory-informed themes. We leveraged Self-Care Theory to understand how patients’ motivation to use the platform and their subsequent use of its features generated perceived value and challenges in achieving this value.

**Results:**

The platform was shown to support patients’ development of core self-care abilities (cognitive, psychosocial, and sociocultural abilities) and self-care behaviors (maintenance, monitoring, management), but it did not provide any support to the development of physiological abilities. Moreover, results indicate important limitations in the way in which the digital platform supported all self-care abilities and behaviors—in particular, self-care management. Hence, while the platform was viewed as valuable overall, patients reported several challenges in effectively using the Chronic Care Platform for self-care.

**Conclusions:**

Digital platforms for chronic disease management can enhance patient self-care by providing valuable resources and support for reinforcing desired behaviors. However, gaps in platform features can limit patients’ ability to comprehensively care for themselves. This study shows that relating platform features to specific dimensions of self-care can help identify missing features, providing a fine-grained understanding of how a given platform is generating positive impacts and how it may be improved to fully support self-care.

## Introduction

There has been a key shift in disease prevalence across the globe over the past several decades. As improvements in the treatment of communicable diseases have led to a global decrease in premature mortality, aging populations are now increasingly affected by one or more noncommunicable diseases, also called chronic diseases [1]. As a result, chronic diseases such as cardiovascular disease, diabetes, and chronic respiratory diseases have become leading causes of death, accounting for over 74% of deaths globally every year [2]. Yet, the burden of chronic diseases can be significantly diminished by reducing their risk factors, which include modifiable behaviors such as tobacco use, unhealthy diet, and lack of exercise. While varied types of interventions can help individuals manage these behaviors, self-management support interventions are both the most frequently used and the most successful in terms of improvements in patient-level outcomes [3].

Information and communication technologies are increasingly used to deliver self-management interventions for chronic disease management [4], as well as a wide variety of related interventions, including medical consultations [5], education, and early detection [6]. Solutions focused on self-management allow patients to play an active role in the management of their disease and facilitate interactions and collaborative care among patients and health care professionals [7]. Moreover, they can be key to scaling up chronic disease interventions in a cost-effective manner [8,9]. Studies of digital interventions often show clinical and health benefits for chronic conditions. For example, a review of 65 systematic reviews and randomized controlled trials showed that interventions using mobile health technology for chronic diseases such as chronic respiratory disease, diabetes, chronic kidney disease, and cardiovascular diseases generally have positive behavioral and clinical effects [10]. Similarly, a meta-analysis of 26 articles consisting of 22 studies with 2645 participants showed that smartphone-based self-management interventions for diabetes patients have positive impacts on self-efficacy, self-care activities, and health-relevant outcomes such as quality of life [11].

Recently, digital platforms offering a multitude of information and communication technology–based interventions for self-management have emerged [12]. These platforms provide wide-ranging support, such as learning resources, personalized self-monitoring, and communication with peers and care professionals. Digital platforms can flexibly adapt and update these interventions through generic hardware, software, and data components [13]. While studies of patients’ experiences with platform-based interventions for chronic disease management show overall positive results, mixed experiences and expectations regarding aspects such as data entry burden, digital literacy, and provider feedback can hinder the adoption and perceived value of these interventions [14,15]. Such ambivalent experiences [14] may result from the wide array of platform features that can generate unexpected impacts, as one feature may reinforce or hinder another. The complexity and flexibility of digital platforms thus necessitate new approaches to understanding their impacts on self-management outcomes, beyond classical approaches such as clinical trials focused on individual interventions.

This study aims to explore how a digital platform can both enable and limit patients suffering from a chronic condition in managing their own health. The context for this study is a Canadian chronic disease management program for people who are at risk of cardiovascular disease or who have suffered from cardiovascular issues. A digital platform, referred to as the Chronic Care Platform in this study, is used to deliver the health program. We use Self-Care Theory [16] to articulate the positive and negative value that patients perceive from the use of the Chronic Care Platform. Self-Care Theory reframes the concept of self-management into several abilities and behaviors that are required for individuals to successfully manage their conditions [17,18]. As a result, we identify important gaps in the way in which the platform enables patients to care for their health. Beyond the Chronic Care Platform, this study illustrates an approach to analyzing the comprehensive interplay of platform features and self-care abilities and behaviors. The results of this study can also be used to support the configuration and design of digital platforms for chronic disease self-management.

## Methods

### Overview

This study adopts a qualitative inquiry approach, which seeks to build an understanding of the world from the experiences, beliefs, and ideas of people while acknowledging that multiple interpretations of reality may coexist [19]. This approach was chosen because it allows for an understanding of patients’ experiences using a digital platform. Semistructured interviewing was chosen as the data collection method as it is useful for gaining an understanding of participants’ perspectives related to a domain of interest while remaining open to exploring other relevant topics during the course of the interview [20]. The study focuses on a single digital platform to enable a richer understanding of the relationship between specific platform features and patient experiences. We sought to recruit patients who had used this platform for at least 1 month to ensure sufficient experience for the study.

We used the SRQR (Standards for Reporting Qualitative Research) reporting guideline when editing this manuscript [21], and its associated checklist is included in Checklist 1.

### Ethical Considerations

The study was conducted in compliance with the Declaration of Helsinki, including anonymizing research participant data throughout the analysis and reporting process, ensuring that participants did not feel any undue pressure to take part in the study nor to answer interview questions, and holding interviews at times and locations that were considered convenient for each participant. Written informed consent was obtained from all participants before data collection. Participants were not compensated. The study protocol and all associated methods were approved by the University of Ottawa’s Office of Research Ethics and Integrity (S-11-19-5244).

### Chronic Care Platform

The study focuses on patients’ experiences with a digital platform referred to as the Chronic Care Platform for the purpose of this study. The platform is used to deliver a chronic disease management program for individuals who have suffered or are at risk of suffering from cardiovascular disease. The program was created and is managed by a hospital providing specialized secondary and tertiary care and had been underway for several years when the study started and remains available. The program targets 2 populations: patients who have received heart disease–related care at this hospital (post discharge) and individuals at risk of experiencing heart disease (prevention). The goal of this program is to help both populations improve their risk factors in order to prevent future life-threatening events.

The Chronic Care Platform is designed to facilitate access to the program and allow the hospital to serve a larger number of individuals. The platform is accessible through a web browser and an app offering features desired by patients to manage their conditions [22]: an online library with varied educational material related to medication, prognosis, and care; a personal health record where patients can record, access, and manage their health information; care and wellness plan; and online rehabilitation support with exercise guidelines. Additionally, the Chronic Care Platform allows scheduling meetings with a health coach for patients who are given access to as part of the program, sharing information with individuals such as family members and physicians as desired by the patient, and interacting with other users through a forum. The platform also supports the development of care and wellness plans, and the tracking and monitoring of varied metrics such as blood pressure, steps, weight, exercise, and mood. It provides visual displays to understand trends related to these metrics (eg, weight gain, daily exercise duration). Information can be tracked through manual entry or by syncing with wearable and health-monitoring devices and apps. As such, the Chronic Care Platform reflects typical digital platforms for self-management.

### Participant Recruitment

Participant recruitment followed a convenience sampling strategy. Patients enrolled in the chronic care management program, delivered through the Chronic Care Platform, were invited to participate in the study. Invitations to participate in the study were sent to platform users by the health care program coordinator using the platform messaging system. The message invited interested patients to contact the researchers directly. Recruitment material was also posted on the walls of the hospital where the chronic care management program was being administered. Patients who were using or had used the Chronic Care Platform for at least 1 month were invited to participate in the study. No additional inclusion or exclusion criteria were used in order to allow the recruitment of participants with varied experiences. However, since participants were recruited within the chronic care management program, they were adults (aged 18 y or older) at risk of heart disease or with chronic heart disease. All interested and eligible participants were recruited for the study since the experiences of any user of the platform were of interest to the study.

### Data Collection

The first author and a research assistant conducted semistructured interviews with the 24 recruited patients. The first author had experience conducting such interviews and provided training to the research assistant prior to starting interviews. They did not have any preexisting relationship or relationship of authority with the participants. Following the same predefined interview protocol for each interview, patients were asked about their motivation for using the platform, how they used the platform, and the resulting value perceived alongside any challenges in realizing this value (Table 1). Each interview concluded with an open-ended question that encouraged patients to share anything they thought to be relevant about the Chronic Care Platform. Nine interviews were conducted in person and the remainder by phone. In-person interviews were held at locations chosen for their convenience by the participants; these locations included private rooms at the hospital delivering the chronic disease management program and public spaces such as coffee shops. Participants chose a space or table that they felt was appropriate for the interview in the latter case.

**Table 1.** Categories and questions asked to participants.

Category	Question
Context	How did you hear about the Chronic Care Platform? Are you using it as part of a postdischarge care plan, for preventing future issues, or both? How long have you been using it?
Motivation	Why did you decide to use the Chronic Care Platform? Do you feel that it allows you to reach certain goals or solve certain problems? Which ones?
Platform use	Who do you interact with through the Chronic Care Platform? How do you use the Chronic Care Platform in order to reach the goals or solve the problems that you stated previously? Which platform content, services, functionalities, or tools do you use?
Perceived value and challenges	How is the Chronic Care Platform or its contents beneficial to you in your everyday life? Have you experienced challenges in using the Chronic Care Platform?
Open	Would you like to share anything else about the Virtual Care platform or your experience with it?

Interviews lasted between 20 and 60 minutes, with an average of 30 minutes. They were audio-recorded with the consent of participants and transcribed verbatim by a research assistant and verified for accuracy by the first author. Any identifying information was removed from the transcripts prior to data analysis, and each participant was labeled with a number (eg, P1). As the number of participant interviews increased, substantial redundancies emerged in reported motivations, usage, and perceptions, indicating that the dataset had reached saturation [23]. However, data saturation was not used as a criterion for concluding data collection, and all recruited participants were interviewed.

### Data Analysis

Template analysis was used to analyze interview data. It is a type of thematic analysis that allows both the inductive identification of themes from data and the deductive application of theory-informed themes [24,25]. Each participant interview was thus first coded inductively using labels that reflected the collected data, and similar codes across participant interviews were then merged into initial themes that reflected broader meanings. This process allowed us to discern emerging patterns of use through recurring themes regarding patients’ motivation for using the platform (eg, “understanding,“ “accessing care”), how the platform was used (eg, “tracking,” “self-educating”), and the kinds of benefits and challenges that patients experienced (eg, “being encouraged,” “irrelevant information”). Two researchers were involved in this process, comparing code application, discussing any discrepancy, and clarifying label and theme definitions.

We then turned to existing theories that could provide a relevant explanation for these patterns. We initially reviewed behavior change theories, including the Theory of Planned Behavior [26] and the Health Belief Model [27]. However, while these theories focus on explaining what may drive individuals’ intended and observed behaviors (eg, the concept of “perceived benefits” in the Health Belief Model), they did not provide sufficient coverage of the emerging themes, nor did they help to achieve the study’s objective. These theories and others that are frequently cited in informatics, such as the Technology Acceptance Model [28], propose concepts that can be used to predict the adoption of healthy behaviors and supporting technology. However, their focus on behavioral intentions does not provide the concepts needed to unpack the behaviors themselves such as the platform uses that were present in our data (eg, “self-educating”). We thus turned to Self-Care Theory [16], which provided a natural fit to our data and emerging themes.

Self-care refers to purposeful actions or activities that are performed deliberately and continuously by an individual to maintain their life, healthy functioning, and well-being [29,30]. These activities can be initiated independently by the individual or in collaboration with health care professionals [30]. The notion of self-care thus aligns with common definitions of self-management [31]. In the context of chronic conditions, self-care is key to empowering people in assuming responsibility for their own health [32]. This understanding of self-care has been formalized in Self-Care Theory, which distinguishes between an individual’s capacity to engage in self-care behavior (self-care abilities), and the actions that can be performed to realize this care (self-care behaviors [17,18,33]).

Self-care abilities encompass 4 groups of factors: cognitive, psychosocial, physiological, and sociocultural [17,18,34] (Textbox 1). While some authors also include demographic factors such as age, we do not include them here as they can be viewed as basic conditioning factors whose impacts are better captured through specific self-care ability domains [34]. Self-care behaviors refer to 3 interrelated processes that are initiated by individuals on their own or in collaboration with health care professionals: self-care maintenance, self-care monitoring, and self-care management [17,18] (Textbox 1).

Textbox 1. **Factors supporting self-care ability and processes of self-care behavior.**

Factors supporting self-care ability are as follows:

1. *Cognitive factors* such as learning skills provide an individual with the cognitive skills needed to fulfill self-care behaviors. They include cognitive function, learning skills, memory, problem-solving and goal-setting skills, awareness and knowledge of health condition, and knowledge of self-care behaviors.
2. *Psychosocial factors*, also referred to as emotional factors, focus on an individual’s feelings, values, and motivations that can influence if and how they will perform self-care. These factors include an individual’s self-esteem, self-discipline ability, personality traits, perceived self-competence, self-efficacy, motivation, and perception that the behavior to be carried out is efficacious.
3. *Physiological factors,* such as psychomotor skills, also referred to as physical factors, impact the ability of an individual to perform needed self-care behaviors. They relate to an individual’s dexterity, psychomotor skills, functional or movement level, as well as their disability or injury status.
4. *Sociocultural factors,* such as an individual’s family system, can enable or constrain one’s self-care behaviors. These contextual factors include an individual’s family system, cultural beliefs and practices, social support, health beliefs and values, available resources, and access to care.

Processes of self-care behavior are as follows:

1. *Self-care maintenance* refers to the selection of appropriate activities to improve well-being or manage health problems. For chronic conditions, these activities often reflect those recommended by health care providers and include lifestyle-related activities such as preparing healthy foods and those related to following a medical regimen such as taking medication as prescribed.
2. *Self-care monitoring* is a process of surveillance of one’s body that aims at allowing an individual to recognize that a change has occurred. In the context of chronic conditions, this process is key to enabling the recognition of health problems through changes in signs or symptoms.
3. *Self-care management* is a process of implementing a treatment in response to changes indicating a deterioration in condition, as well as evaluating the effectiveness of that treatment. The treatment may involve an independent modification of self-care maintenance activities or a change requiring consultation with a health care provider. For patients with a chronic condition, the preference is for empowering them and their care partners (eg, spouse) to independently select and engage in self-care activities.

The Self-Care Theory concepts (in italics in Textbox 1) were deductively applied to identified themes and their data by 2 researchers, comparing code application, discussing any discrepancy, and clarifying code definition. Relationships between concepts were also identified when present in the data (eg, “Cognitive abilities—supporting—self-care maintenance”). Each interview was then coded by 1 researcher, with frequent revision of code application by the second author and inputs on the interpretations by the third author. Both the inductive and deductive phases of data analysis were informed by the research team’s training and experience in digital technology and platforms developed and used in health care contexts (LL, MdR) as well as in cardiovascular health and prevention (KAM). Their combined viewpoints allowed the team to identify meaningful information that could have been glossed over from any single perspective and to compare varied assumptions and interpretations.

## Results

### Overview

A total of 33 potential participants contacted the researchers. Two of them were excluded because they had used the platform less than 1 month, 6 participants did not respond to follow-up communications, and 1 withdrew from the study prior to being interviewed. A sample of 24 participants consisting of 9 (37.5%) women and 15 (62.5%) men was thus recruited. Among them, 79% (19/24) participants were part of the postdischarge program and 21% (5/24) participants belonged to the prevention program. Twelve of 24 (50%) participants were receiving or had received advice from a health coach provided by the program for the first 6 months of use. The frequency at which they used the platform varied from occasional to daily, with 42% (10/24) accessing the platform on a weekly or daily basis. All participants had been using the platform for at least 1 month, 12 (50%) of them having been regular users for 1 year or more. Table 2 provides more detailed information on participant characteristics.

**Table 2.** Participant characteristics (N=24).

Participant number	Sex	Program	Health coach	Frequency of use	Length of use
P1	Male	Postdischarge	No	Daily	<3 mo
P2	Female	Postdischarge	No	Daily	3‐6 mo
P3	Female	Prevention	Yes	>Weekly	>1 y
P4	Male	Postdischarge	Initially	Rarely	>1 y
P5	Female	Postdischarge	Yes	>Weekly	3‐6 mo
P6	Female	Postdischarge	Initially	>Monthly	>1 y
P7	Male	Postdischarge	No	Daily	>1 y
P8	Male	Postdischarge	Initially	Monthly	>1 y
P9	Male	Post-discharge	No	>Year	>1 y
P10	Male	Postdischarge	No	Weekly	6 mo to 1 y
P11	Female	Prevention	Initially	Never	6 mo to 1 y
P12	Male	Postdischarge	No	>Monthly	6 mo to 1 y
P13	Female	Prevention	Initially	Monthly	6 mo to 1 y
P14	Male	Postdischarge	No	Daily	>1 y
P15	Male	Postdischarge	Initially	>Weekly	>1 y
P16	Male	Postdischarge	Yes	>Weekly	<3 mo
P17	Male	Postdischarge	Yes	Rarely	6 mo to 1 y
P18	Male	Postdischarge	No	Never	6 mo to 1 y
P19	Female	Prevention	No	>Monthly	a^a^
P20	Male	Postdischarge	No	>Weekly	>1 y
P21	Male	Postdischarge	Initially	a^a^	>1 y
P22	Male	Postdischarge	Yes	>Weekly	<3 mo
P23	Female	Postdischarge	No	Monthly	>1 y
P24	Female	Prevention	No	Rarely	>1 y

a “a” indicates that the participant did not answer.

### Platform Support to Self-Care

In our analysis, we investigated how motivations for self-care translated into platform use, which subsequently not only generated perceived value but also highlighted challenges for patients in effectively using the platform to achieve the desired goals. Overall, we found that the platform offered support for all self-care abilities and behaviors except physiological abilities (Table 3). The platform, however, shows limitations for self-care management support and important challenges in its support to all self-care abilities and behaviors.

**Table 3.** Summary of the identified patients’ patterns of use of the Chronic Care Platform for each self-care ability and behavior.

Self-Care Theory concepts	Motivations	Platform features (and external resources)	Platform use	Perceived value	Challenges
Cognitive abilities	Understanding how to change behaviors and improve health using a trusted source of information	Health-related generic information Health-related questionnaires Personalized care plans, workbooks, and reports Space for manual medical data entry and goals (External: websites containing health-related information)	Accessing health-related information and prescribed activities (eg, exercise) Entering personal goals and medical information Filling out surveys, consulting workbooks, and reports	Feeling educated, inspired, and able to achieve goals	Missing personal tailoring and more advanced information Not understanding platform functioning or where to find desired content Difficulty in processing information due to disease-related cognitive disability
Psychosocial abilities	Finding positive validation and reaffirming motivation to change behavior	Information on mental health Reward system providing automated positive feedback based on tracked measures	Educating oneself about anxiety and stress Consulting reward points and automated congratulations	Feeling reassured, motivated, and empowered	Not perceiving tangible benefit from reward system
Physiological abilities	No data	No data	No data	No data	No data
Sociocultural abilities	Connecting with other patients and health care providers Feeling safe, watched over	Discussion forum Scheduling and emailing feature Data sharing feature (External: social apps, in-person visits to general practitioner, in-person interactions with family members and peers)	Reading content written by other patients on the forum Communicating and sharing data with health coach Adding chosen individuals to account, allowing them to view data and send reminders	Interacting and learning from peers Getting support from a health coach	Missing true social interaction Sharing data with external physicians is difficult
Self-care maintenance	Changing lifestyle and developing healthy behaviors, routines, and solutions	Exercise recommendations and descriptions (External: classes and equipment at local gym)	Initiating lifestyle changes and exercising independently	Integrating new behaviors into lifestyle	Lacking proactive recommendations/guidance
Self-care monitoring	Efficiently measuring own activity, food intake, and health data Understanding cause and effect among metrics	Manual data entry Data importation from compatible tracking devices Dashboard showing trends over time (External: tracking devices and apps)	Monitoring health-related measures and symptoms Viewing trends on platform dashboard	Having relevant insights into progress, reinforcing willingness to continue self-care efforts	Entering data and synchronizing with external devices are challenging
Self-care management	Assessing whether lifestyle changes are effective	Dashboard showing trends over time	Viewing trends on platform dashboard	Visualizing progress over time	Unable to self-assess if health status requires physician input No alerts going to health care provider

### Patterns of Use for Self-Care Abilities

#### Cognitive Abilities

Patients are looking for reliable information and insight into how to change behavior. To do so, they combine generic health information, such as articles related to diet, exercise, and lifestyle, and patient-specific information with interactive resources such as exercise workbooks. As a result, they feel educated, inspired, and reminded to change behavior:

*I think one of the biggest things is that it’s a great reminder to me. I was reading something on diabetes, now I’m well versed on diabetes but it never hurts to be reminded.*

[P3]

Patients also feel connected, able to set goals, and structure their lifestyle to achieve them. However, some patients were disappointed by the generic and repetitive nature of available information, especially patients looking for more expert information:

*I would like to see more [...content...] in the library...that pointed directly to me...because there’s a lot of associations or references...which doesn’t really apply to me.*

[P7]

This led some patients to use external, invalidated information rather than the platform:

*I’m active online, so I get...Dr. I can’t remember who,...and they offer advice - regular advice online, and so I find other - I’m bombarded with all sorts of information that seems to come at me.*

[P8]

#### Psychosocial Abilities

Patients seek positive validation and reaffirming motivation to change behavior. Patients educate themselves about how their mental health can impact their motivation and remain accountable and motivated through automated feedback provided by the platform:

*When you log something they send you messages right away, keep up the good work. Positive reinforcement which is really great*.

[P2]

As a result, they feel reassured that they are not alone in this process and feel empowered and motivated to continue. However, some patients felt that actual rewards would have motivated them more:

*Because I have 6600 points and I don’t know what those points are for. I’m not getting a prize or a cup of coffee at the end*.

[P2]

#### Sociocultural Abilities

Three dimensions of sociocultural abilities were highlighted through data analysis: availability of resources, social support, and access to care. The first dimension concerns the convenience of using the platform to avoid using personal resources such as time and money to receive in-person support and care:

*We only have one car between the two of us and we are going different ways all the time...and then the parking is atrocious there. Just getting a spot and then once you get it’s expensive.*

[P4]

This motivation is thus not related to a specific platform functionality.

The second dimension, social support, is motivated by the desire to connect with others. The platform supports this dimension through the forum, which enables reading content written by other patients and interacting with them, and the data sharing feature allows patients to add individuals to their environment, typically family members:

*[...] so data from my [external apps and devices] would find a way onto the platform, which I found extremely useful for me for keeping a record, but also for those who were sharing that information, and I had my physiotherapist, my wife, the health coach*.

[P7]

As a result, patients feel reassured and enjoy interacting and learning from peers. However, few participants reported using the forum, and some participants felt that real social interactions were missing, particularly in relation to the forum:

*It would have been nice to say hey everyone, let’s be buddies or whatever, because the social aspect would be nice.*

[P10]

The third dimension, access to care, is related to the desire to get advice from health care providers by sharing information with them, as well as to feel safe and cared for:

*[...] part of my rehabilitation was to change my lifestyle and I felt that the platform gave me a virtual link to somebody...the health coach to make sure that I was on the right track. It just gave me some reassurance that somebody was interested, besides my wife and my family of course*.

[P4]

Patients used meeting and scheduling features to interact with the health coach but were sometimes disappointed that the data on the platform were not used by their physicians, either because they are unwilling or unaware:

*Okay I had a six month interview with [a physician external to the intervention] about 2‐3 weeks ago. I...showed him the blood pressure history—but...he didn’t seemed to be overly interested*.

[P5]

Relatedly, participants were uncertain who would benefit from the effort of inserting data.

#### Physiological Abilities

The collected data did not generate any results regarding participants’ physiological abilities. Hence, when participants were asked about their motivation for using the Chronic Care Platform, the way in which they used it, and the value and challenges that they perceived through that use (Table 1), participants did not refer to elements that could be categorized as physiological or physical abilities that allowed them to carry out self-care behaviors, or hindered them from doing so.

### Patterns of Use for Self-Care Behaviors

#### Self-Care Maintenance

Participants want to change their lifestyle and develop healthy behaviors, routines, and solutions. The platform offers exercise recommendations and descriptions to do so. Patients use these features, at times in combination with external resources such as a local gym, to exercise independently and initiate lifestyle changes:

*I prefer to be on my own, do my own workouts and updating the system. To me that was a natural*.

[P4]

As a result, participants experience benefits by integrating new behaviors into their lifestyle. However, some participants expressed the desire for more proactive recommendations and guidance.

#### Self-Care Monitoring

Patients are interested in efficiently measuring their own activity, food intake, and health data, and in understanding cause and effect among metrics. To do so, they both manually enter data such as weight, food intake, and blood sugar levels and connect external tracking devices such as smart watches to the platform. They also make use of nonconnected tracking devices and diaries. They are thus able to monitor an array of health-related measures and symptoms, and to view resulting trends through the platform dashboard:

*There really is something about being able to look back over the month or the six months and see the graphs...something about that was just - I found it really encouraging.*

[P6]

This provides them with relevant insights on their health, which reinforces their willingness to continue their efforts toward self-care:

*[...] you can’t sort of fool yourself in terms of your diet...It sort of reinforces your whole diet program in a sense that you know if you can stick to a certain calorie intake you are going to lose weight which is positive reinforcement*.

[P5]

However, participants perceived the need to manually enter data as burdensome and experienced challenges in importing data from external trackers.

#### Self-Care Management

The importance of this pattern is the ability to assess whether lifestyle changes are effective. The only related platform support is a dashboard feature that patients use to visualize trends related to tracked metrics; it is also used for self-care monitoring, which enables one to see the progress over time. However, participants expressed negative perceptions related to their inability to judge if trends reflected improvements or deterioration in health status and when clinical interventions may be appropriate:

*[...] it’d be nice to have a metric to kind of know, oh hey, you’re running your life ragged and you’re in danger of having a heart attack, because a lot of this is prevention*.

[P10]

Several participants were also unsure if these metrics were being monitored by anyone else:

*Does a medical doctor look at if the blood pressure readings are bad? Say, hey somebody better flag this guy and get him in. I don’t know.*

[P4]

### Feedback Loops Among Platform Features and Self-Care

Data show that self-care abilities and behaviors impact each other through the platform’s patterns of use. For example, the informational material supporting the development of cognitive and psychosocial activities (eg, educational material related to stress, recipes) generates learning, which is key to integrating effective lifestyle changes as part of self-care maintenance. Another example is that the platform’s tracking features provide incentive (psychosocial abilities) to enter or import and view health-related data (self-care monitoring), which in turn provides positive reinforcement (psychosocial abilities), supports interactions with the coach (sociocultural abilities), and enables patients to integrate behavioral change into their current lifestyle (self-care maintenance). The results of these interactions are thus positive loops among platform features, abilities, and behaviors.

Conversely, challenges in using the platform and missing features were found to hinder the realization of positive loops among self-care components. For example, a participant (P16) stopped tracking their data since the results were stable and they did not see any reason to continue. Putting this decision in the context of the very limited features provided by the platform for self-care management—features that would include, among others, the ability for users to assess the effectiveness of their lifestyle changes—points to negative impacts of gaps within the Chronic Care Platform that could affect patients’ health outcomes over time.

## Discussion

### Contributions

This study leverages Self-Care Theory [16,18] to analyze patients’ patterns of use—related patient motivations, platform features, patient activities, and resulting perceptions of value—of the Chronic Care Platform. This provides a fine-grained understanding of how platform features can support self-care abilities and behaviors. Such explanations go beyond the insights that can be gained from exploring patients’ willingness or intention to use a platform using the Theory of Planned Behavior and the Health Beliefs Model [4,35], or from overall perceptual measures such as usability, ease of use, and usefulness of digital health technology [13,35].

Understanding the role of the Chronic Care Platform in facilitating self-care provides context to clinical studies that are evaluating the behavioral and clinical impacts of the program being delivered by the platform. An earlier 12-month study of the program conducted with 478 participants showed significant improvements in participants’ cardiovascular disease risk—with an average reduction of 93 (19.5%) in Framingham risk score, improvements in anxiety, perceived stress, physical activity, and more [36]. The program, at that time, offered telephone-based virtual coaching, but was not being delivered using the Chronic Care Platform. A more recent study evaluating the program for postpartum women, delivered using the Chronic Care Platform, showed similar results [37]. A total of 190 women taking part in the 12-month study showed a 102 (53.7%) reduction in Framingham scores alongside a 43 (22.6%) average reduction in Lifetime Risk Scores, significant decrease in body weight, hypertension, and other clinical outcomes, increase in physical activity levels, and healthy eating.

These results contribute to the body of knowledge on the impact of digital health interventions on chronic disease self-management and the digital health technology features that are needed to successfully deliver them. For example, 2 systematic studies, one on platform-based interventions for self-management of chronic diseases [38] and the other on the efficacy of smartphone-based digital health interventions [39], arrived at similar results. That is, both reviews found that the majority of included studies showed significant improvements in behavioral outcomes such as physical activity. However, the results were mixed for clinical outcomes such as blood pressure and body weight. While several included studies showed positive outcomes (sometimes with minimal effects, however), the majority failed to demonstrate statistically significant improvements.

Better understanding of the features and role of the digital health technologies used to deliver such interventions may help to understand variability in clinical outcomes. Digital health technology includes various software and devices used to process and exchange health information on the internet, including websites, wearable devices, smartphones, and more [40]. Their most common functionalities relate to communication (eg, messaging), self-monitoring (eg, tracking of health measures), tailored self-care support (eg, personalized goals and reminders), self-care education (eg, links to reference material), care planning (eg, personal care plan), and community forums [39,40]. The Chronic Care Platform reflects the proposition for digital health platforms to integrate these functionalities into an environment that is easily accessible through various devices. A recurring observation across studies of digital health technology and platforms is that despite their obvious potential for self-managing chronic diseases, they are yet to meet patients’ expectations regarding tailored, personalized support with an acceptable combination of automated and human guidance [38,39,41]. The challenge in meeting these expectations is not solely technological, in particular with the growing availability of large datasets and artificial intelligence (AI) capabilities that can be used to truly personalize care plans, prevention recommendations, alerts, dietary recommendations, and more [42,43]. The results of this study, in line with other studies of digital health technology [38,39], rather emphasize that the gap lies in using these features in a manner that creates value and generates patient engagement and retention.

The use of Self-Care Theory also helps to situate commonly identified patient needs. For example, while other studies have also found that patients express the need for personalized and relevant information when using digital solutions to support chronic disease management [4,44], they do not justify why such information is important. Self-Care Theory helps to understand that personalized information is needed to develop the cognitive abilities required by patients to care for themselves. Another example is the request for features facilitating the organization of health community activities to increase social engagement [13], which can be understood as supporting the development of sociocultural abilities in Self-Care Theory. As new technologies such as AI become integrated into self-care digital platforms [44], this understanding is key to differentiating needs that are core to chronic disease management from those that are specific to AI-based systems.

**Figure 1.**
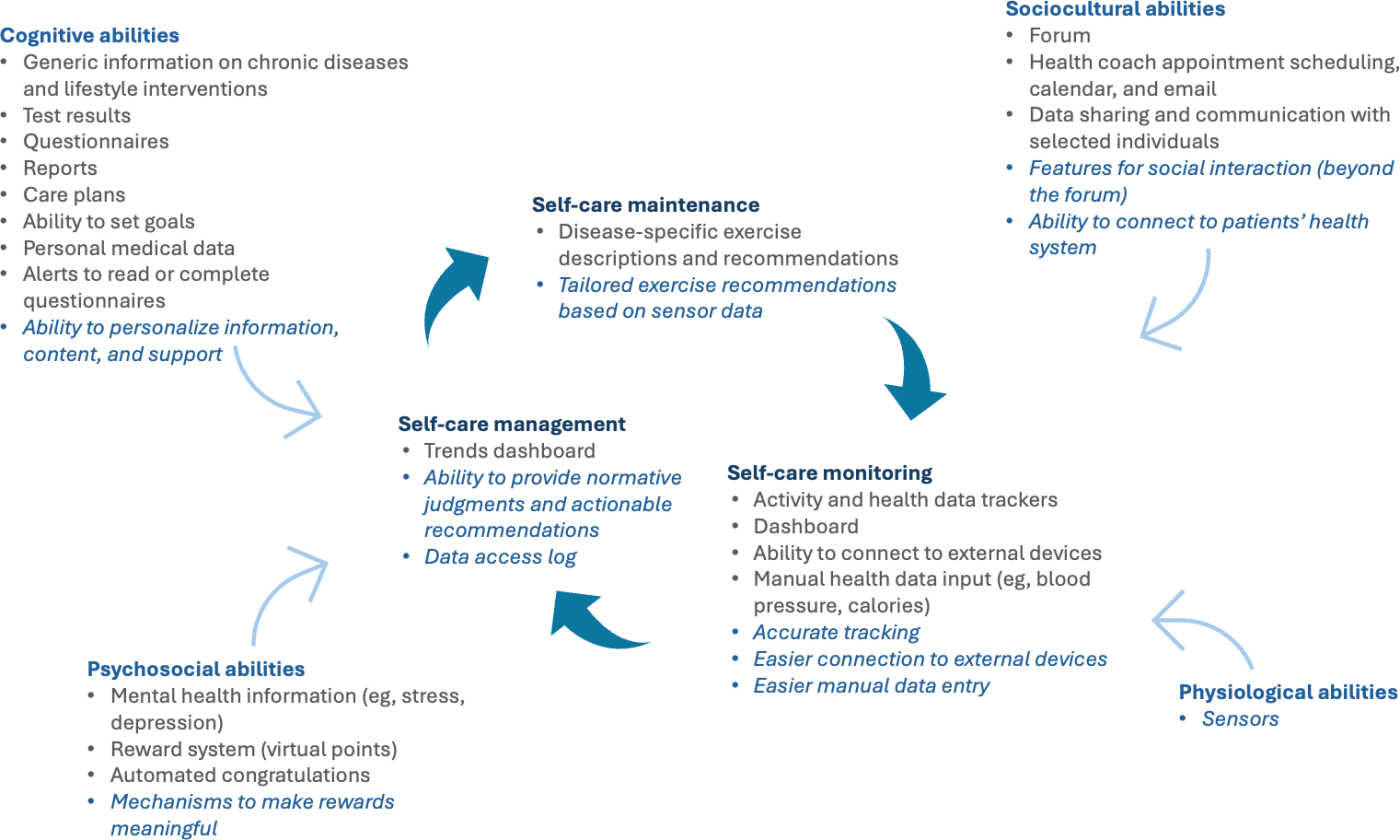
Existing and missing platform features for self-care abilities and behaviors. Bullet points identify key features of the chronic care platform for each self-care ability and behavior. Bullet points in italics refer to missing features in the chronic care platform.

Moreover, the use of Self-Care Theory to make sense of this study’s results helps to identify gaps in the Chronic Care platform’s features. Figure 1 identifies key features of the platform for each self-care ability and behavior, as well as missing features that could address the challenges experienced by participants. A key gap, the lack of platform support for the development of physiological self-care abilities, can be observed across studies of digital platforms for chronic disease management. This points to a missed opportunity to support chronic disease management more comprehensively, for example, by making platforms interoperable with movement-focused sensors that could assess a patient’s initial state and lead to tailored exercise recommendations. Such features would also help to improve the platform’s limited support for Self-Care Management, which shows important gaps in enabling normative judgment (eg, assessing if a trend is healthy or unhealthy) and providing actionable recommendations (eg, recommendation to call one’s physician). The integration of AI capabilities could address several of these gaps by leveraging sensor-generated and other patient data to better support all self-care behaviors, for example, facilitating meal tracking through image recognition (self-care monitoring) [43]; predicting and alerting the patient about low blood glucose events (self-care management) [42]; and providing tailored nutritional intervention messages to prevent future events (self-care maintenance). However, patients’ reticence in using features such as AI chatbots would need to be mitigated [41,45].

Other gaps pertain to the quality or the way in which functionalities have been designed. For example, the platform does contribute to the development of psychosocial abilities through information and feedback, but could have more impact if the feedback had real-life implications for participants. Similarly, while the ability to share data with selected individuals generated positive value perceptions, the forum only partially supported the development of sociocultural abilities. In both of these examples, the gaps are not functional—information is provided as planned, the forum functions as intended—but rather relate to the inability of available features to meet participants’ expectations.

An apparent disconnect between patient expectations and what is offered through the platform was also apparent in relation to features that facilitate interactions with a health coach, which were positively evaluated by many participants. More generally, results showed that several participants sought reassurance and feedback from health care providers through the platform, which is in line with similar studies [41]. However, virtual care for chronic disease management is often seen as a way to increase efficiency, whereby scarce human resources can be leveraged to care for a greater patient population by transferring self-care responsibilities to the patient while maintaining or improving patient outcomes [46]. Providing features that enable normative judgment and personalized, actionable recommendations may partially address patients’ need to be reassured in their progress. Beyond such automated support, the benefits of added human support should be evaluated against costs when creating digital health interventions for self-management of chronic conditions [38].

Nevertheless, when making design choices for digital health platforms in this context, it is important to account for the ripple effects of individual features across several self-care abilities and behaviors. For example, the support provided by a health coach improves sociocultural abilities for self-care, but these improved abilities in turn affect almost all other abilities and self-care behaviors. Conversely, the lack of mechanisms to make rewards meaningful (gap in psychosocial abilities support) could diminish a patient’s motivation to track their progress (self-care monitoring behavior), hindering their ability to detect and react to changes in health status (self-care management behavior). The same functionalities are now typically integrated across digital health technology and platforms for self-care [39,40], and these functionalities are expected by patients [41]. The challenge of designing new solutions hence does not lie, in the choice or creation of discrete functionalities, but rather in the interlinking of these functionalities to trigger virtuous cycles of self-care abilities and behaviors. This can be achieved in varied ways, for example, by codesigning digital health platforms with target end users to understand if and how features support desired patterns of use and by ensuring software and hardware interoperability (eg, automated data import from self-tracking wearable devices) to facilitate seamless flow among self-care behaviors.

### Limitations

Despite its contributions, the study has several limitations. The study is based on a single digital platform within a single health care setting (cardiovascular disease), which could limit the applicability of the results to other platforms. This limit was however mitigated by analyzing data using a relevant theory that provides generalizable explanations for self-care. As such, the patterns of use identified in this study and resulting recommendations for fully realizing patient value are likely to apply to other digital platforms aiming to support self-care for patients suffering from other chronic diseases. The small number of participants (n=24) also limits the generalizability of results, while being in line with similar qualitative studies [41]. Nevertheless, future research on digital health platforms for chronic disease self-management would benefit from adopting a mixed methods approach with quantitative analysis of representative datasets. Methodological limitations related to analysis and interpretation of the data in qualitative studies were mitigated by involving 3 researchers in the coding process and by iteratively moving between interpretations and data to ensure that findings are grounded in the data as collected. The use of multiple researchers in data analysis corresponds to investigator triangulation, while the consideration of alternative theories as a source of concepts for the deductive analysis phase corresponds to theoretical triangulation [47]. Both types of triangulation used in the study aimed at increasing the trustworthiness of study results.

### Conclusions

This study examined patients’ patterns of use of a digital platform to understand how the platform both enabled and limited their ability to self-manage their chronic disease. Digital platforms are increasingly being used in the context of chronic disease self-management, without a full understanding of how their features may influence patients’ experiences and generate desired health outcomes. We contribute to this issue by analyzing what motivates patients to use a chronic self-care platform, how they subsequently use the platform’s features, and how this use generates both positive and negative values. The resulting patterns of use are interpreted as supporting the development of required self-care abilities and the realization of core self-care behaviors [16,18]. Experienced challenges lead to the identification of gaps in platform features that limit patients’ ability to fully care for themselves. This study thus provides a fine-grained understanding of how a given platform can generate positive impacts and how it may be improved to fully support self-care.

## Supplementary material

10.2196/71875 Checklist 1 Reporting items and their location based on the Standards for Reporting Qualitative Research (SRQR) and its associated checklist.

## Abbreviations

AI: artificial intelligence
SRQR: Standards for Reporting Qualitative Research

## References

[1] James SL, Abate D, Abate KH (2018). Global, regional, and national incidence, prevalence, and years lived with disability for 354 diseases and injuries for 195 countries and territories, 1990–2017: a systematic analysis for the Global Burden of Disease Study 2017. The Lancet.

[2] Noncommunicable diseases. World Health Organization.

[3] Reynolds R, Dennis S, Hasan I (2018). A systematic review of chronic disease management interventions in primary care. BMC Fam Pract.

[4] Shen H, van der Kleij R, van der Boog PJM (2022). Digital tools/eHealth to support CKD self-management: a qualitative study of perceptions, attitudes and needs of patients and health care professionals in China. Int J Med Inform.

[5] Randine P, Sharma A, Hartvigsen G, Johansen HD, Årsand E (2022). Information and communication technology-based interventions for chronic diseases consultation: scoping review. Int J Med Inform.

[6] Abqari U, van ’t Noordende AT, Richardus JH, Isfandiari MA, Korfage IJ (2022). Strategies to promote the use of online health applications for early detection and raising awareness of chronic diseases among members of the general public: a systematic literature review. Int J Med Inform.

[7] Nikayin F, De Reuver M, Itälä T (2013). Collective action for a common service platform for independent living services. Int J Med Inform.

[8] Gaziano TA, Galea G, Reddy KS (2007). Scaling up interventions for chronic disease prevention: the evidence. Lancet.

[9] Kyaw TL, Ng N, Theocharaki M, Wennberg P, Sahlen KG (2023). Cost-effectiveness of digital tools for behavior change interventions among people with chronic diseases: systematic review. Interact J Med Res.

[10] Fan K, Zhao Y (2022). Mobile health technology: a novel tool in chronic disease management. Intell Med.

[11] Aminuddin HB, Jiao N, Jiang Y, Hong J, Wang W (2021). Effectiveness of smartphone-based self-management interventions on self-efficacy, self-care activities, health-related quality of life and clinical outcomes in patients with type 2 diabetes: a systematic review and meta-analysis. Int J Nurs Stud.

[12] Breeman LD, Keesman M, Atsma DE (2021). A multi-stakeholder approach to eHealth development: promoting sustained healthy living among cardiovascular patients. Int J Med Inform.

[13] Park M, Bui LK, Jeong M (2021). ICT-based person-centered community care platform (IPC3P) to enhance shared decision-making for integrated health and social care services. Int J Med Inform.

[14] Taylor ML, Thomas EE, Vitangcol K (2022). Digital health experiences reported in chronic disease management: an umbrella review of qualitative studies. J Telemed Telecare.

[15] van de Vijver S, Hummel D, van Dijk AH (2022). Evaluation of a digital self-management platform for patients with chronic illness in primary care: qualitative study of stakeholders’ perspectives. JMIR Form Res.

[16] Denyes MJ, Orem DE, Bekel G (2001). Self-care: a foundational science. Nurs Sci Q.

[17] Sidani S, Doran DI (2014). Development and validation of a self-care ability measure. Can J Nurs Res.

[18] Riegel B, Jaarsma T, Strömberg A (2012). A middle-range theory of self-care of chronic illness. ANS Adv Nurs Sci.

[19] Liamputtong P (2019). Handbook of Research Methods in Health Social Sciences.

[20] Adeoye‐Olatunde OA, Olenik NL (2021). Research and scholarly methods: semi‐structured interviews. J Am Coll Clin Pharm.

[21] O’Brien BC, Harris IB, Beckman TJ, Reed DA, Cook DA (2014). Standards for reporting qualitative research: a synthesis of recommendations. Acad Med.

[22] Bally ELS, Cheng D, van Grieken A (2023). Patients’ perspectives regarding digital health technology to support self-management and improve integrated stroke care: qualitative interview study. J Med Internet Res.

[23] Saunders B, Sim J, Kingstone T (2018). Saturation in qualitative research: exploring its conceptualization and operationalization. Qual Quant.

[24] King N, Brooks J, Tabari S, Ciesielska M, Jemielniak D (2018). Qualitative Methodologies in Organization Studies.

[25] Cheng VWS, Piper SE, Ottavio A, Davenport TA, Hickie IB (2021). Recommendations for designing health information technologies for mental health drawn from self-determination theory and co-design with culturally diverse populations: template analysis. J Med Internet Res.

[26] Ajzen I, Kuhl J, Beckmann J (1985). Action Control From Cognition to Behavior.

[27] Rosenstock IM (1974). The health belief model and preventive health behavior. Health Educ Monogr.

[28] Rahimi B, Nadri H, Lotfnezhad Afshar H, Timpka T (2018). A systematic review of the technology acceptance model in health informatics. Appl Clin Inform.

[29] Orem DE, Taylor SG, Renpenning KM (2001). Nursing: Concepts of Practice.

[30] Sidani S, Doran DM (2011). Nursing Sensitive Outcomes The State of the Science.

[31] Richardson J, Loyola-Sanchez A, Sinclair S (2014). Self-management interventions for chronic disease: a systematic scoping review. Clin Rehabil.

[32] Riegel B, Dunbar SB, Fitzsimons D (2021). Self-care research: where are we now? Where are we going?. Int J Nurs Stud.

[33] Hartweg DL (1991). Dorothea Orem: Self-Care Deficit Theory.

[34] Burks KJ (1999). A nursing practice model for chronic illness. Rehabil Nurs.

[35] Chen Y, Xu Q (2022). The willingness to use mobile health and its influencing factors among elderly patients with chronic heart failure in Shanghai, China. Int J Med Inform.

[36] Prince SA, Reid RD, Pipe AL, McDonnell LA (2017). An evaluation of CardioPrevent: a technology-enabled, health-behavior change program for the global reduction of cardiovascular risk. Curr Opin Cardiol.

[37] Quansah DY, Martin N, McDonnell L (2025). Association between a technology-enabled postpartum program and cardiovascular disease risk reduction in women with prior hypertensive disorders of pregnancy. O G Open.

[38] Tighe SA, Ball K, Kensing F, Kayser L, Rawstorn JC, Maddison R (2020). Toward a digital platform for the self-management of noncommunicable disease: systematic review of platform-like interventions. J Med Internet Res.

[39] Suresh S, Bhardwaj S, Rodrigues HC (2025). Smartphone-based digital health interventions: a comprehensive systematic review of efficacy for cardiovascular and cerebrovascular outcomes. J Med Syst.

[40] Wannheden C, Åberg-Wennerholm M, Dahlberg M (2022). Digital health technologies enabling partnerships in chronic care management: scoping review. J Med Internet Res.

[41] Tighe SA, Ball K, Kayser L, Kensing F, Maddison R (2022). Qualitative study of the views of people living with cardiovascular disease, and healthcare professionals, towards the use of a digital platform to support cardiovascular disease self-management. BMJ Open.

[42] Shomali M, Mora P, Aleppo G (2024). The critical elements of digital health in diabetes and cardiometabolic care. Front Endocrinol (Lausanne).

[43] Lee YB, Kim G, Jun JE (2023). An integrated digital health care platform for diabetes management with AI-based dietary management: 48-week results from a randomized controlled trial. Diabetes Care.

[44] Wang B, Asan O, Zhang Y (2024). Shaping the future of chronic disease management: insights into patient needs for AI-based homecare systems. Int J Med Inform.

[45] Wu PF, Summers C, Panesar A, Kaura A, Zhang L (2024). AI hesitancy and acceptability-perceptions of AI chatbots for chronic health management and long COVID support: survey study. JMIR Hum Factors.

[46] Carter HE, Wallis S, McGowan K (2023). Economic evaluation of an integrated virtual care programme for people with chronic illness who are frequent users of health services in Australia. BMJ Open.

[47] Dootson S (1995). An in-depth study of triangulation. J Adv Nurs.

