# Supplementary material for: Understanding How a Digital Platform for Chronic Disease Management Can Enable and Limit Patient Self-Care: Qualitative Study
Source: J Med Internet Res. 2026 May 14;28:e71875. doi: 10.2196/71875 (PMC13175445; doi:10.2196/71875)
Supplement: Checklist 1 [file jmir-v28-e71875-s001.docx]

**SRQR Checklist**

| **No.** | **Topic** | **Item** |
| --- | --- | --- |
| **Title and abstract** | | |
| S1 | Title | Understanding how a digital platform for chronic disease management can enable and limit patient self-care: A qualitative study |
| S2 | Abstract | *Background*. A growing segment of the population requires ongoing care and support for managing their chronic diseases. Digital platforms for self-management are rapidly emerging to meet this need, but patients’ experience with these platforms varies significantly. This may be due to the complexity and flexibility of digital platforms, where the wide array of available features can generate unexpected impacts.  *Objective.* This study aims to explore how a digital platform can both enable and limit patients suffering from a chronic disease in managing their own health.  *Methods*. We conducted semi-structured qualitative interviews with patients to better understand their experience of using a digital platform for self-managing their chronic disease. Patients that had been using a digital platform referred to as the Chronic Care Platform for at least one month were invited to participate. Twenty-four patients were recruited and interviewed in-person or by phone. Collected data were analyzed using template analysis, which is a type of thematic analysis that allows inductive identification of themes from data and deductive application of theory-informed themes. We leveraged Self-Care Theory to understand how patients’ motivations to use the platform and their subsequent use of its features generated perceived value and challenges in achieving this value.  *Results*. The platform was shown to support patients’ development of core self-care abilities (cognitive, psychosocial and sociocultural abilities) and self-care behaviours (maintenance, monitoring, management), but did not provide any support to the development of physiological abilities. Moreover, results indicate important limitations in the way in which the digital platform supported all self-care abilities and behaviours, in particular self-care management. Hence, while the platform was viewed as valuable overall, patients reported several challenges in effectively using the Chronic Care Platform for self-care.  *Conclusions*. Digital platforms for chronic disease management can enhance patient self-care by providing valuable resources and support for reinforcing desired behaviours. However, gaps in platform features can limit patients’ ability to comprehensively care for themselves. The study shows that relating platform features to specific dimensions of self-care can help to identify missing features, providing a fine-grained understanding of how a given platform is generating positive impacts and how it may be improved to fully support self-care. |
| **Introduction** | | |
| S3 | Problem formulation | While studies of patients’ experiences with platform-based interventions for chronic disease management show overall positive results, mixed experiences and expectations regarding aspects such as data entry burden, digital literacy, and provider feedback can hinder the adoption and perceived value of these interventions. Such ambivalent experiences may result from the wide array of platform features that can generate unexpected impacts, as one feature may reinforce or hinder another. The complexity and flexibility of digital platforms thus necessitate new approaches to understanding their impacts on self-management outcomes, beyond classical approaches such as clinical trials focused on individual interventions. |
| S4 | Purpose or research question | The purpose of this study was to explore how a digital platform can both enable and limit patients suffering from a chronic condition to manage their own health. |
| S5 | Qualitative approach and research paradigm | The study was conducting from an interpretive research paradigm, which seeks to build an understanding of the world from the experiences, beliefs and ideas of people while acknowledging that multiple interpretations of reality may co-exist [1]. There are many variations of this perspective, including some that adhere to a subjectivist epistemology (there are multiple ways of knowing) while adopting a more realist ontology (there is, probably, an objective truth) [2].  This study belongs to the latter. Indeed, on one hand, it builds its understanding from participants’ perception and used an inductive approach as a first step to data analysis, but then deductively applied codes representing core concepts of Self-Care Theory [3].  Self-Care Theory was chosen for this study because it enabled the research team to articulate the positive and negative value that patients perceive from the use of a platform for chronic care self-management. Self-Care Theory reframes the concept of self-management into several abilities and behaviours that are required for individuals to successfully manage their conditions.  This approach was relevant for the study because it allowed an in-depth exploration of participants’ perceptions and reported use of the platform while establishing linkages between participants’ experiences, specific platform features and abilities and behaviours known to be important for successful chronic disease self-management. |
| S6 | Researcher characteristics and reflexivity | The first and second authors approached the study from their training and research experience in information systems and digital platforms developed and used in healthcare contexts. The third author brought clinical expertise in cardiology, chronic care and digital interventions. The first author conducted some of the interviews but did not have any pre-existing relationship or relationship of authority with participants. The third author was involved in the management of the digital platform for chronic disease self-management being studied, but they did not participate in data collection.  The combined viewpoints of the research team enabled its members to explore collected data using varied foci. It also allowed them to identify meaningful information that could have been glossed over from any single viewpoint. Moreover, research team discussions allowed comparing varied interpretations and assumptions, which spurred reflexivity. |
| S7 | Context | The context for this study is a Canadian chronic disease management program for people who are at risk of cardiovascular disease, or who have suffered from cardiovascular issues. The program was created and is managed by a hospital providing specialized secondary and tertiary car, had been underway for several years when the study started and remains available. A digital platform, referred to as the Chronic Care Platform in this study, is used to deliver the health program. The Chronic Care Platform is meant to facilitate access to the program and to allow the hospital to serve a larger number of individuals.  Focusing on the Chronic Care Platform was relevant for this study because it reflects typical digital platforms for self-management of chronic diseases. |
| S8 | Sampling strategy | The study relied on a convenience sampling strategy, hence all interested and eligible participants were recruited. This strategy was relevant for the study since the recruitment targeted patients enrolled in the chronic care management program delivered through the Chronic Care Platform, and the experience of any of those participants was of interest to the study.  Invitations to participate in the study were sent to platform users by the health care program coordinator using the platform messaging system. The message invited interested patients to contact the researchers directly. Recruitment material was also posted on the walls of the hospital where the chronic care management program was being administered. Patients who were using or had used the Chronic Care Platform for at least one month were invited to participate in the study. No additional inclusion or exclusion criteria were used in order to allow the recruitment of participants with varied experiences. However, since participants were recruited within the chronic care management program, they were adults (aged 18 years or older) at risk of heart disease or living with heart disease. All interested participants who met eligibility criteria were included.  Data saturation was not used as a criterion for concluding data collection, and all recruited participants were interviewed. However, as the number of participant interviews increased, substantial redundancies emerged in reported motivations, usage, and perceptions, indicating that the dataset had reached saturation [4]. |
| S9 | Ethical issues pertaining to human subjects | The study was conducted in compliance with the Declaration of Helsinki including by anonymizing research participant data throughout the analysis and reporting process, ensuring that participants did not feel any undue pressure to take part in the study nor to answer interview questions, and by holding interviews at times and locations that were considered convenient for each participant. Written informed consent was obtained from all participants before data collection. Participants were not compensated. The study protocol and all associated methods were approved by the University of Ottawa’s Office of Research Ethics and Integrity (S-11-19-5244). |
| S10 | Data collection methods | The first author and a research assistant conducted semi-structured interviews with recruited participants. The first author had experience conducting such interviews and provided training to the research assistant prior to starting interviews.  Following a pre-defined interview protocol, patients were asked about their motivation for using the platform, how they used the platform, and the resulting value perceived alongside any challenge in realizing this value. Each interview concluded with an open-ended question that encouraged patients to share anything they thought to be relevant about the Chronic Care Platform. This semi-structured interview approach was selected to focus data collection on information relevant to answer the research question while allowing participants to discuss topics that they felt were important in their specific situation. |
| S11 | Data collection instruments and technologies | Interviewers followed the same interview guide for each interview. The main questions asked to participants are presented in Table 1. Probes were also developed to further elicit information sharing by participants (e.g., for the question “Have you experienced challenges in using the Chronic Care Platform?”, the probing question “Have these challenges impacted the way in which you use the platform?” was also asked when participants answered with a "yes” or “no”.)  Nine interviews were conducted in person and the remainder by phone. In-person interviews were conducted in a location chosen for its convenience by the participants; these locations included private rooms at the hospital delivering the chronic disease management program and public spaces such as coffee shops. Participants chose a space or table that they felt were appropriate for the interview in the latter case. Interviews lasted between 20 and 60 minutes with an average of 30 minutes. They were audio recorded with the consent of participants. |
| S12 | Units of study | 33 potential participants contacted the researchers. 2 of them were excluded because they had used the platform less than one month, 6 participants did not respond to follow-up communications, and 1 withdrew from the study prior to being interviewed. A sample of 24 participants consisting of 9 women and 15 men was thus recruited. The number of participants was considered sufficient for a qualitative study and reflects similar studies.  19 participants (79%) were part of the post-discharge program, and 5 participants (21%) belonged to the prevention program. 12 participants (50%) were receiving or had received advice from a health coach provided by the program for the first six months of use. The frequency at which they used the platform varied from occasional to daily, with 10 participants (42%) accessing the platform on a weekly or daily basis. All participants had been using the platform for at least one month, 12 of them (50%) having been regular users for one year or more. Table 2 provides more detailed information on participant characteristics. |
| S13 | Data processing | Audio recordings of interviews were transcribed verbatim by a research assistant and verified for accuracy by the first author. Any identifying information was removed from transcripts prior to data analysis, and each participant was labeled with a number (e.g., P1). |
| S14 | Data analysis | Template analysis was used to analyze interview data. Template analysis is a type of thematic analysis that allows both the inductive identification of themes from data, and the deductive application of theory-informed themes [5,6]. Each participant interview was thus first coded inductively using labels that reflected the collected data, and similar codes across participant interviews were then merged into initial themes that reflected broader meanings. This process allowed us to discern emerging patterns of use through recurring themes regarding patients’ motivation for using the platform (e.g., ‘understanding’, ‘accessing care’), how the platform was used (e.g., ‘tracking’, ‘self-educating’), and the kinds of benefits and challenges that patients experienced (e.g., ‘being encouraged’, ‘irrelevant information’). A research assistant as well as the first and second authors were involved in this process, comparing code application, discussing any discrepancy, and clarifying label and theme definitions.  We then turned to existing theories that could provide a relevant explanation for these patterns. We initially reviewed behaviour change theories including the Theory of Planned Behaviour [7] and the Health Belief Model [8]. Howev[9]er, while these theories focus on explaining what may drive individuals’ intended and observed behaviours (e.g., the concept of ‘perceived benefits’ in the Health Belief Model), they did not provide sufficient coverage of the emerging themes, nor did they help to achieve the study’s objective. These theories and others frequently cited in informatics, such as the Technology Acceptance Model [10], propose concepts that can be used to predict the adoption of healthy behaviours and supporting technology. However, their focus on behavioral intentions does not provide the concepts needed to unpack the behaviours themselves such as the platform uses that were present in our data (e.g., ‘self-educating’). We thus turned to Self-Care Theory [3], which provided a natural fit to our data and emerging themes.  Self-Care Theory concepts (in italics in Textbox 1) were deductively applied to identified themes and their data by two researchers, comparing code application, discussing any discrepancy, and clarifying code definition. Relationships among concepts were also identified when present in the data (e.g., “Cognitive abilities ---supporting --- self-care maintenance”). Each interview was then coded by the first author, with frequent revision of code application by the second author and input on the results by the third author. |
| S15 | Techniques to enhance trustworthiness | Potential bias in individual viewpoints was mitigated by involving three researchers in the coding process and by iteratively moving between interpretations and data to ensure that findings are grounded in the data as collected.  The use of multiple researchers in data analysis corresponds to investigator triangulation, while the consideration of alternative theories as a source of concepts for the deductive analysis phase corresponds to theoretical triangulation [11]. Both types of triangulations increase the validity of study results.  We documented the articulation and application of codes throughout the analysis process, as well as interpretive comments made by individual researchers. Annotated documents were used as a basis for discussion among research team members. Evolving versions of coded data were kept, forming an audit trail contributing to analytical rigour. |
| **Results** | | |
| S16 | Synthesis and interpretation | In our analysis, we investigated how motivations for self-care translated into platform use, which subsequently generated perceived value but also highlighted challenges for patients in effectively using the platform to achieve desired goals. Overall, we found that the platform offered support for all self-care abilities (cognitive, psychological, sociocultural) except physiological abilities. All self-care behaviours (maintenance, monitoring, management) were also shown to be supported. The platform, however, shows limitations for self-care management support and important challenges in its support to all self-care abilities and behaviours.  Results also showed the presence of feedback loops among platform features and self-care. For example, the informational material supporting the development of cognitive and psychosocial activities (e.g., educational material related to stress, recipes) generates learning, which is key to integrating effective lifestyle changes as part of self-care maintenance. Conversely, challenges in using the platform and missing features were found to hinder the realization of positive loops among self-care components. The result of these interactions are thus positive and negative loops among platform features and self-care abilities and behaviours.  A synthesis of the results are provided in Table 3.  Interpretation of the results focusing on gaps in the Chronic Care platform’s features are provided in the Discussion section and in Figure 1, which identifies key features of the platform for each self-care ability and behaviour, as well as missing features that could address the challenges experienced by participants. |
| S17 | Links to empirical data | Participant quotes are provided for each self-care ability and behaviour in the Results section. |
| S18 | Integration with prior work, implications, transferability, and contributions to the field | Results are shown to provide a fine-grained explanation of how platform features can support self-care abilities and behaviours. Such explanations go beyond the insights that can be gained from exploring patients’ willingness or intention to use a platform using the Theory of Planned Behaviour and the Health Beliefs Model [12,13], or from overall perceptual measures such as usability, ease of use, and usefulness of digital health technology (e.g., [13,14]).  They also help to understand variability in clinical outcomes of studies focusing on the impacts of digital health interventions. Indeed, the results of this study emphasize that the common observation that digital health technology and platforms do not meet patients’ expectations is not rooted in technological limitations but in using these features in a manner that creates value and generates patient engagement and retention. Designing digital health technology in a manner that provides more comprehensive support for self-care abilities and behaviours is thus a promising avenue to increase patient satisfaction with these solutions.  While the study focused on a single digital health platform, results may be transferable to similar technology and programs. To increase transferability, the setting of the study is described in the Chronic Care Platform subsection of the Methods section, through a detailed presentation of both the platform being studied and the program that it delivers. Clinical studies of the program and comparisons of the Chronic Care Platform and its features with similar digital health technology and platforms are also provided in the Discussion section. |
| S19 | Limitations | Despite its contributions, the study has several limitations. The study is based on a single digital platform within a single healthcare setting (cardiovascular disease), which could limit the applicability of the results to other platforms. This limit was however mitigated by analyzing data using a relevant theory that provides generalizable explanations for self-care. As such, the patterns of use identified in this study and resulting recommendations for fully realizing patient value is likely to apply to other digital platforms aiming to support self-care for patients suffering from other chronic diseases. The small number of participants (n=24) also limits the generalizability of results, while being in line with similar qualitative studies [38]. Nevertheless, future research on digital health platforms for chronic disease self-management would benefit from adopting a mixed-methods approach with quantitative analysis of representative data sets. Methodological limitations related to analysis and interpretation of the data in qualitive studies was mitigated by involving three researchers in the coding process and by iteratively moving between interpretations and data to ensure that findings are grounded in the data as collected. |
| **Other** | | |
| S20 | Conflicts of interest | Authors declare no conflicts of interest. |
| S21 | Funding | This work was supported by a Telfer School of Management Research Grant, which was secured by the first author. |
